# Infiltration and persistence of lymphocytes during late-stage cerebral ischemia in middle cerebral artery occlusion and photothrombotic stroke models

**DOI:** 10.1186/s12974-017-1017-0

**Published:** 2017-12-15

**Authors:** Yan Feng, Shiwei Liao, Changjuan Wei, Dongmei Jia, Kristofer Wood, Qiang Liu, Xiaoying Wang, Fu-Dong Shi, Wei-Na Jin

**Affiliations:** 1 Department of Neurology, Tianjin Neurological Institute, Tianjin Medical University General Hospital, Tianjin, 300052 China; 2 Center for Neuroinflammation, Beijing TianTan Hospital, Beijing, 100070 China; 3 Department of Neurology, Barrow Neurological Institute, St. Joseph’s Hospital and Medical Center, Phoenix, 85013 AZ USA; 4 Neuroprotection Research Laboratory, Departments of Radiology and Neurology, Massachusetts General Hospital, Harvard Medical School, Boston, 02129 MA USA

**Keywords:** Inflammation, Lymphocytes, Ischemic stroke, Photothrombosis, Transient middle cerebral artery occlusion

## Abstract

**Background:**

Evidence suggests that brain infiltration of lymphocytes contributes to acute neural injury after cerebral ischemia. However, the spatio-temporal dynamics of brain-infiltrating lymphocytes during the late stage after cerebral ischemia remains unclear.

**Methods:**

C57BL/6 (B6) mice were subjected to sham, photothrombosis, or 60-min transient middle cerebral artery occlusion (MCAO) procedures. Infarct volume, neurodeficits, production of reactive oxygen species (ROS) and inflammatory factors, brain-infiltrating lymphocytes, and their activation as well as pro-inflammatory cytokine IFN-γ production were assessed. Brain-infiltrating lymphocytes were also measured in tissue sections from post-mortem patients after ischemic stroke by immunostaining.

**Results:**

In mice subjected to transient MCAO or photothrombotic stroke, we found that lymphocyte infiltration persists in the ischemic brain until at least day 14 after surgery, during which brain infarct volume significantly diminished. These brain-infiltrating lymphocytes express activation marker CD69 and produce proinflammatory cytokines such as IFN-γ, accompanied with a sustained increase of reactive oxygen species (ROS) and inflammatory cytokines release in the brain. In addition, brain-infiltrating lymphocytes were observed in post-mortem brain sections from patients during the late stage of ischemic stroke.

**Conclusion:**

Our results demonstrate that brain-infiltration of lymphocytes persists after the acute stage of cerebral ischemia, facilitating future advanced studies to reveal the precise role of lymphocytes during late stage of stroke.

**Electronic supplementary material:**

The online version of this article (10.1186/s12974-017-1017-0) contains supplementary material, which is available to authorized users.

## Background

Peripheral lymphocytes infiltrating ischemic brain regions orchestrate inflammatory responses, catalyze neuronal death, and worsen clinical outcomes of acute ischemic stroke (AIS) [1–3]. Previous studies have focused on the dynamics and function of brain-infiltrating lymphocytes during the acute stage of brain ischemia, i.e., from hours to days after onset [4–10]. In acute ischemic stroke, the majority of infiltrating lymphocyte subsets can damage neural structures and exacerbate stroke outcome [11–15]. For example, γδ T, CD8^+^ T, and NK cells contribute to acute brain injury after stroke onset [16, 17], while regulatory T and B cells are reported to be protective [18]. However, little is known about the presence and functional status of brain-infiltrating lymphocytes during the late stage of cerebral ischemia.

Transient middle cerebral artery occlusion (MCAO) and photothrombotic ischemia are two commonly used murine models to study neuroinflammation in stroke. Although the post-ischemic inflammation and injury have been well documented during the early stage of ischemia in these two models, the features of cellular immune responses have not been adequately studied during the late stage.

In this study, we determined the dynamics, activation, and cytokine production profiles of brain-infiltrating lymphocytes up to 14 days after photothrombotic and MCAO in mice during which brain infarct volume has significantly diminished, in conjunction with post-mortem human brain tissues from patients with ischemic stroke. Our results suggest that lymphocyte responses can persist in the brain at least for weeks after cerebral ischemia.

## Methods

### Mice

Male C57BL/6 (B6, H2^b^) mice and Rag2^−/−^γc^−/−^mice (H2^b^) were purchased from Taconic (Oxnard, CA, USA). Mutant mice were backcrossed to the B6 background for at least 12 generations. Mice were maintained under specific pathogen-free conditions, and used at 10–12 weeks of age. They were housed with no more than five animals per cage under standardized light-dark cycle conditions with ad libitum access to food and water. For all experiments, age-matched male littermates were used. All animal experiments were performed in accordance with the ARRIVE (Animal Research: Reporting in vivo Experiments) guidelines. All procedures were approved by Animal Care and Use Committees of the Barrow Neurological Institute and Tianjin Neurological Institute.

### Transient middle cerebral artery occlusion procedure

Adult male 10- to 12-week-old mice were subjected to 60 min focal cerebral ischemia produced by transient intraluminal occlusion of the middle cerebral artery (MCAO) as described previously [19–23]. MCAO was performed under anesthesia induced by inhalation of 3.5% isoflurane and maintained by inhalation of 1.0–2.0% isoflurane in 70% N_2_O and 30% O_2_. Body temperature was monitored throughout surgery with a rectal probe and maintained at 37.0 ± 0.5 °C using a heating pad (Sunbeam, Neosho, MO, USA). Cerebral blood flow was monitored for 5 min both before and after MCAO, and immediately before and after reperfusion with a laser Doppler probe (model P10, Moor Instruments, Wilmington, DE, USA). A monofilament made of 6–0 nylon with rounded tip was used to induce focal cerebral ischemia for 60 min by occlusion of the right middle cerebral artery. After 60 min of MCAO, the occluding filament was withdrawn gently back into the common carotid artery to allow reperfusion. Mice were excluded upon death or non-satisfactory cerebral blood flow (CBF) during occlusion or 10 min after reperfusion. Mice that had a residual CBF < 20% of pre-ischemic levels throughout the ischemic period and CBF recovery > 80% within 10 min of reperfusion were used in the study. Sham-operated mice were subjected to the same surgical procedure, but the filament was not advanced far enough to occlude the middle cerebral artery. 7T-MRI was used to determine infarct volume 1–14 days after MCAO [19]. In the MCAO model, the mortality rate was 13.4% (13 of total 97) and exclusion rate was 18.5% (13 of total 97 for inadequate reperfusion, 5 of total 97 for criteria limitations set for the mNSS scoring system).

### Photothrombotic stroke procedure

Photothrombotic occlusion was performed as previously reported [24]. Mice subjected to photothrombotic surgery were anesthetized by inhalation of 3.5% isoflurane and maintained by inhalation of 1.0–2.0% isoflurane in 70% N_2_O and 30% O_2_. Mice were injected intraperitoneally with rose Bengal at a dose of 150 mg/kg (Sigma Aldrich, St. Louis, MO, USA) 5 min prior to illumination. After receiving Rose Bengal, mice were placed into a stereotaxic apparatus (Stoelting Co, Wood Dale, IL, USA). The bregma was identified and the end of a fiber optic cable with a diameter of 4 mm was placed over the top of the skull rostrocaudally centered on and approximately 2 mm lateral to the bregma. Five minutes after injection of the Rose Bengal solution, a cold light source (Schott KL 1600 LED, Elmsford, NY, USA) with a green bandpass filter (Thor Labs, Newton, NJ, USA) was turned on and the skull was illuminated for 20 min. Once the region had been illuminated for 20 min, the mouse was removed from the stereotaxic apparatus and the incision was sutured and sterilized. Sham-operated mice were subjected to the same surgical procedure without Rose Bengal injection. Mice were excluded upon death or non-satisfactory modified Neurological Severity Score [mNSS score < 6 or above a score of 13 at 24 h after photothrombosis (prior to treatment) were excluded]. In the photothrombotic stroke model, the mortality rate was 6% (5 of total 84) and exclusion rate was 8.3% (7 of total 84 non-satisfactory mNSS score).

### Behavior assessment

Behavioral tests were performed at days 1, 3, 7, and 14 after ischemic stroke as we previously described and published reports [19, 20, 25–28]. The modified Neurological Severity Score (mNSS) test consisted of motor, sensory, reflex, and balance assessments. The rating scale was as follows: A score of 13–18 indicates severe injury, 7–12 indicates moderate injury, and 1–6 indicates mild injury. Following surgery, each mouse was assessed on a scale from 0 to 18 after recovery from the stroke procedure. Mice with score < 6 or above a score of 13 at 24 h post surgery (prior to treatment) were not included in the study. In all experiments, 12 mice were excluded due to criteria limitations set for the mNSS scoring system.

For the adhesive removal test, an adhesive patch (0.35 × 0.45 cm) was applied to the forelimb contralateral to the ischemic hemisphere [29]. The mouse was then put into a cage. Time taken to contact and remove adhesive tape was recorded with 120 s being the time limit. The Corner turning test was used to assess sensorimotor and postural asymmetries [30]. All mice tested were allowed to enter a corner with an angle of 30° which required the subject to turn either to the left or the right to exit the corner. This was repeated and recorded ten times, with at least 30 s between trials, and the percentage of right turns out of total turns was calculated. The ability of a mouse to respond to a vibrissae-elicited excitation by forward movement of its forelimb was evaluated with the forelimb placing test, as previously described [22]. Briefly, animals held by their trunk were positioned parallel to a table top and slowly moved up and down, allowing the vibrissae on one side of the head to brush along the table surface. Refractory placements of the impaired (left) forelimb were evaluated and a score was calculated as number of successful forelimb placements out of ten consecutive trials. The rotarod test was performed as previously reported [31]. Mice from sham or stroke groups were placed on an accelerating rotating rod. The speed was increased from 4 to 40 rpm (the acceleration rate to 20 rpm/min) within 5 min. Mice were tested three times daily with a break of at least 5 min between tests. The latency to fall off the rotating rod was recorded by a blinded investigator [31].

### Neuroimaging

Infarct size of both ischemic stroke models was assessed with a 7T small animal MRI (Bruker Daltonics Inc., Billerica, MA, USA) with a 72 mm linear transmitter coil and a mouse surface receiver coil for mouse brain imaging, as we previously described [19, 32]. Mice were under anesthesia by inhalation of 3.5% isoflurane and maintained by inhalation of 1.0–2.0% isoflurane in 70% N_2_O and 30% O_2_ by a face mask. During MRI scanning (10 min per mouse), the animal’s respiration was continually monitored by a small animal monitoring and gating system (SA Instruments, Stoney Brook, NY, USA) via a pillow sensor positioned under the abdomen. Mice were placed on a heated circulating water blanket (Bruker, Billerica, MA) to maintain normal body temperature (36–37 °C). Axial 2D multi-slice T2-weighted images of brain with fat-suppressed Rapid Acquisition with Relaxation Enhancement (RARE) sequence (TR = 4000 ms, effective TE = 60 ms, number of average = 4, FOV = 19.2 mm × 19.2 mm, matrix size = 192 × 192). The ten gradient echoes were acquired for T2*-mapping by using a Multislice Gradient Echo sequence (MGE, TR = 120 ms, echo time = 3.0, 6.0, 9.0, 12.0, 15.0, 18.0, 21.0, 24.0, 27.0, 30.0 ms, field of view = 35.0 × 35.0 mm, matrix = 128 × 128, slice thickness = 1.0 mm, flip angle 80°). The MRI data were analyzed using ImageJ software (National Institutes of Health, Bethesda MD, USA).

The live bioluminescence images were captured to detect reactive oxygen species (ROS) generation in the brain, using the Xenogen IVIS200 imager (Caliper LifeSciences, Hopkinton, MA, USA) [19, 32, 33]. ROS production was assessed using luminol as we and others previously described [22, 23, 26, 33]. Briefly, mice were injected i.p. with 200 mg/kg luminol (Invitrogen, Carlsbad, CA, USA). At 10 min after luminol injection, bioluminescence images were captured. A region of interest tool was used to define and measure the chemiluminescent intensity within the brain. Data were collected as photons per second per cm^2^ using Living Image software (Caliper Life Sciences, Hopkinton, MA, USA).

### Cell isolation, labeling, and passive transfer

Splenocytes of C57BL/6 mice were isolated using red cell lysis buffer (BD Biosciences) as we previously described [19, 34]. For the SPIO-Molday ION Rhodamine-B (MIRB) labeling assay, lymphocytes were sorted using flow cytometry, and cultured with MIRB (BioPhysics Assay Laboratory, Inc., Worcester, MA, USA) at a concentration of 25 μg/ml, in RPMI culture medium with 10% FBS (Invitrogen, Grand Island, NY, USA), l-glutamine (2 mM), IL-2 (10 μg/ml), penicillin (100 U/ml), and streptomycin (0.1 mg/ml) at 5% CO_2_/37 °C, as we previously described [20]. After MIRB incubation for 24 h, cells were centrifuged (400 g for 5 min) and washed twice with PBS to remove extracellular MIRB. Further, 2 × 10^7^ MIRB-labeled lymphocytes in 200 μl PBS were then injected via the tail vein into Rag2^−/−^γc^−/−^ recipient mice. Immediately after transfer, the Rag2^−/−^γc^−/−^ recipients were subjected to sham, 60 min MCAO, or photothrombosis surgeries.

### Flow cytometry

The lymphocytes for FACS assessment were isolated from spleens or brains as we previously described [19, 32, 34]. In brief, splenocytes were isolated using red cell lysis buffer (BD Biosciences). For brain mononuclear cells isolation, after perfusion with cold PBS, the whole brain was dissected, cut into small pieces, and digested in 1 mg/ml Collagenase IV in DMEM at 37 °C for 30 min to obtain the single cells. Single cells were resuspended in 70% percoll and transferred into 15 ml tubes, then overlaid with 37% percoll. The mononuclear cells were harvested after centrifuging at 1800 rpm for 20 min at RT (without brake). Antibodies were labeled with one of the following fluorescent tags: fluorescein isothiocyanate (FITC), phycoerythrin (PE), PerCP-Cy5.5, allophycocyanin (APC), PE-Cy7, or APC-Cy7. The following antibodies were used in this study: CD3 (145-2C11, 553066, BD Biosciences, San Jose, CA, USA), CD4 (RM4-5, 552775, BD Biosciences, San Jose, CA, USA), CD8 (53-6.7, 557654, BD Biosciences, San Jose, CA, USA), NK1.1 (PK136, 551114, BD Biosciences, San Jose, CA, USA), B220 (RA3-6B2, 553093, BD Biosciences, San Jose, CA, USA), IFN-γ (XMG1.2, 505814, Biolegend, San Diego, CA, USA), CD69 (H1.2F3, 104508, Biolegend, San Diego, CA, USA). Flow cytometric measurements were performed on a FACSAria (BD Biosciences, San Jose, CA, USA) and analyzed using FACSDiva and Flowjo 7.6 software (Informer Technologies, Ashland, OR, USA).

### Immunostaining

The immunostaining was performed as we previously described [19, 25, 26, 32, 35, 36]. Briefly, mice were euthanized and whole brain tissue was removed after perfusion, fixed in 4% paraformaldehyde, and then dehydrated with 15 and 30% sucrose. Whole brains were embedded in OCT for preparation of frozen sections. The frozen slices of 30 μm thickness were blocked in 5% goat or donkey serum for 1 h at room temperature. Tissue sections were incubated with primary antibodies against mouse CD4 (MT310, sc-19,641; Santa Cruz Biotechnology, Dallas, Texas, USA), CD8 (ab4055, Abcam, Cambridge, MA, USA), NKp46 (MAB2225, R&D systems, Minneapolis, MN, USA) at 4 °C overnight, and then incubated with appropriate fluorochrome-conjugated secondary antibodies. Nuclei were co-stained with 4′,6-diamidino-2-phenylindole (DAPI; Abcam, Cambridge, MA, USA). For human brain tissue staining, primary antibodies against human CD4 (MAB379-100, R&D systems, Minneapolis, MN, USA), CD8 (MAB1509, R&D systems, Minneapolis, MN, USA), and NKp46 (MAB1850, R&D systems, Minneapolis, MN, USA) were used. Images were acquired on a fluorescence microscope (Olympus, model BX-61, Center Valley, PA, USA) [19, 32, 34]. After immunostaining, positive cell numbers were counted in the every tenth tissue section through the entire tissue block. The number of positive cells in brain sections was expressed as a percentage of immunolabeled cells relative to the total number of cells assessed by DAPI [20, 37]. Image analysis was performed using ImageJ software (National Institutes of Health).

### ELISA

Cytokines in brain homogenates were measured using a customized ELISA kit (SA Biosciences, Valencia, CA, USA). Protein homogenates were extracted from mouse brain using the Halt Protease Inhibitor Cocktail kit (Thermo Fisher Scientific, Fremont, CA, USA) and centrifuged at 13,000 rpm for 20 min at 4 °C, and supernatants were collected. Cytokine levels were detected at 1:20 dilutions according to the manufacturer’s instructions, and reactions analyzed at a wavelength of 450 nm using a 96-well microplate reader (Model 680; Bio-Rad Laboratories, Hercules, CA, USA).

### Human brain tissue

Paraffin-embedded brain tissue sections of stroke patients were obtained from the Sun Health Research Institute (Sun City, AZ) and Department of Pathology, Ohio State University (Columbus, OH). Among the ten human samples used in this study, five tissues were obtained from patients who died within 7–14 days after stroke onset (male, 2; female, 3). The locations of stroke lesions were within the cortical areas supplied by the middle cerebral artery. The tissue was collected from the middle cerebral artery field of perfusion. Five control sections were from patients who died from nonneurological diseases (trauma, 1; kidney failure, 1; gastric cancer, 1; liver cancer, 2) (male, 2; female, 3), and the location of selected tissue sections was matched with stroke patients. All included patients have no acute myocardial infarction, heart failure, autoimmune disease, hematological system disease, or any infection before stroke at the time of death. Patients with ischemic stroke and controls did not differ significantly for mean age at death (stroke patients, 76.2 ± 7 years; controls, 79.5 ± 9.2 years, mean ± SEM; *p* > 0.05, Student’s *t* test). Brain tissues were collected within 4 h after death.

### Statistical analyses

Power analysis and sample size calculations were performed using SAS 9.1 software (SAS Institute Inc. Cary, NC, USA). The experimental design was based on previous publications with similar mechanistic studies [19, 32, 34, 38, 39]. The exclusion criteria are described in the individual method section. Randomization was based on the random number generator function in Microsoft Excel software. All results were analyzed by investigators blinded to different groups. Data are presented as the means ± s.e.m. Statistical significance was determined by the two-tailed unpaired Student’s *t* test for two groups, one-way analysis of variance (ANOVA) followed by Tukey post-hoc test for three or more groups, or two-way ANOVA accompanied by Bonferroni post hoc test for multiple comparisons. Values of *p* < 0.05 were considered significant. All statistical analyses were performed using Prism 6.0 software (GraphPad, San Diego, CA, USA).

## Results

### Evaluation of ischemic damages over time in photothrombotic stroke and MCAO model

**Fig. 1:**
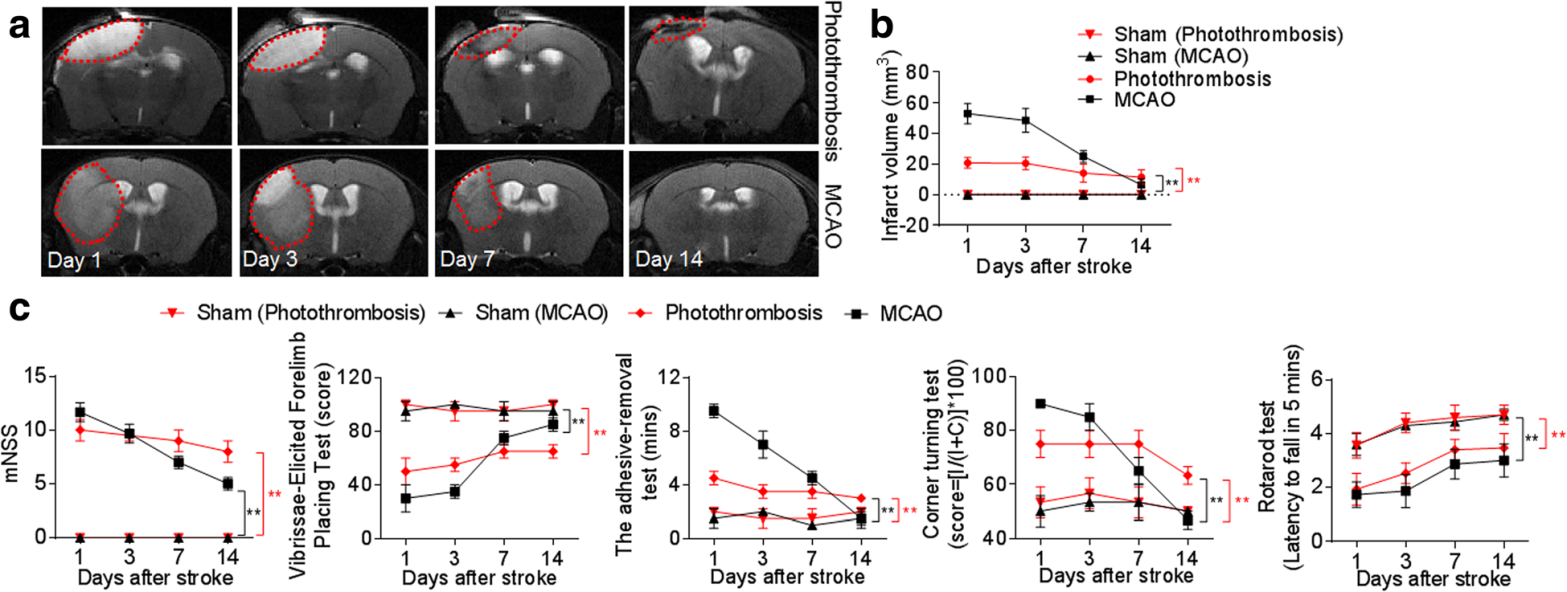
Brain infarct volume and neurological deficits in mice subjected to 60 min MCAO or photothrombosis. C57BL/6 (B6) mice were subjected to sham, photothrombosis, or 60 min MCAO operation. At day 1, 3, 7, or 14 after surgery, mice were subjected to MR imaging and neurological assessment. **a** Representative MRI images show time course of infarct volume (outlined in red) in mice subjected to sham, photothrombosis, or MCAO. **b** Line graphs show the quantification of infarct volume of photothrombosis and MCAO mice at indicated time points after surgery. **c** Cumulative data illustrate the indicated neurological assessments (including mNSS score, the forelimb placing test, adhesive-removing test, corner turning test, and rotarod test) of photothrombosis and MCAO mice from day 1 to day 14 after surgery. *n* = 10 mice per group. Error bars represent s.e.m.; **p < 0.01, sham vs. stroke group (photothrombosis/MCAO) by two-way ANOVA

**Fig. 2:**
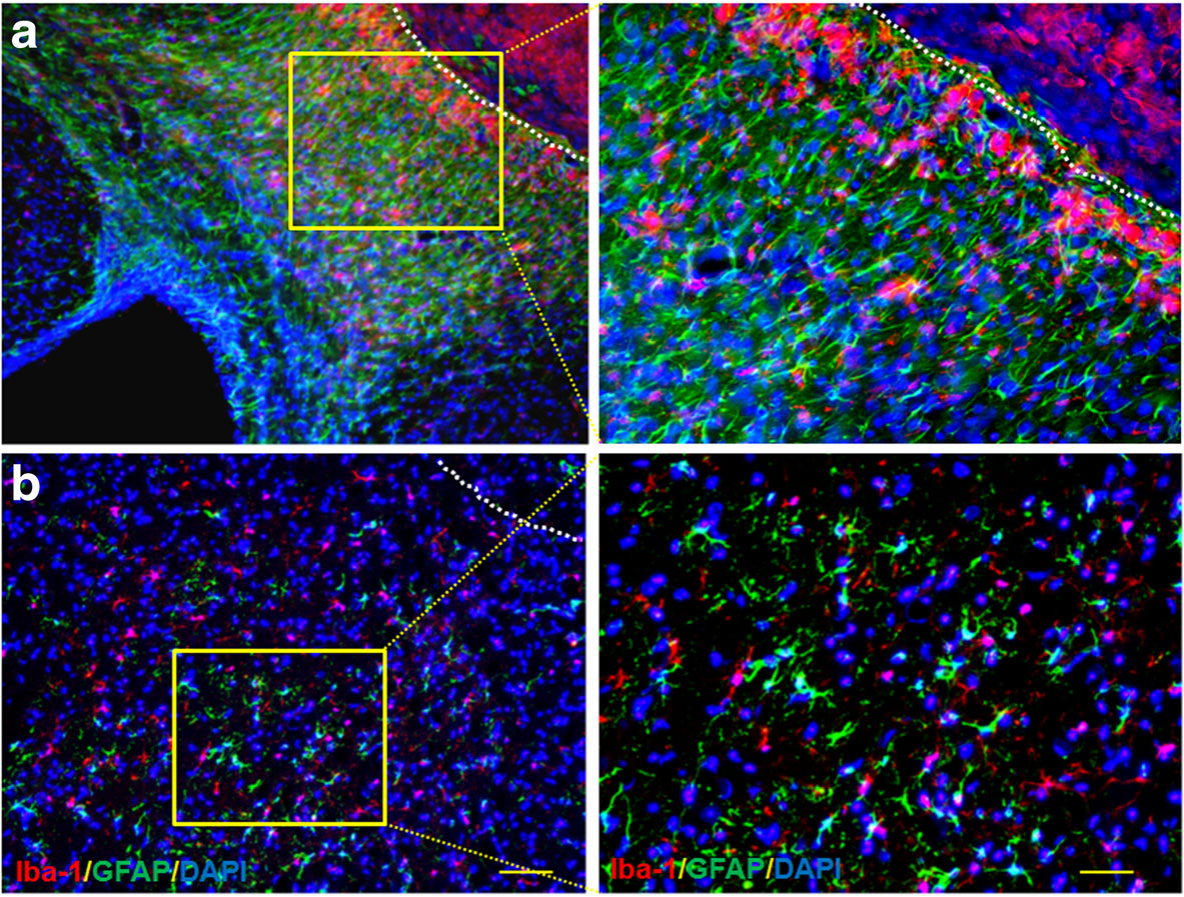
Stroke lesion diminished at day 14 after brain ischemia in mice. Immunostaining of brain sections showing the lesion in the middle of infarction and perilesional area (separated by dashed line) at 14 day after photothrombotic stroke (**a**) and MCAO (**b**). Scale bars: 100 μm (left), 40 μm (right)

7T-MRI T2-w imaging was used to measure the infarct lesion over time (day 1, 3, 7, and 14) after ischemic stroke. The ischemic lesion was evident at 1–3 days both in photothrombosis and MCAO, featured by heterogeneous hyperintense signals on T2-w images (Photothrombosis vs. sham, *n* = 10 per group, *p* < 0.01; MCAO vs. sham, *n* = 10 per group, *p* < 0.01; Fig. 1a, b). Decline of hyperintense signals was found after day 7 on T2-w images both in MCAO and photothrombosis mice. Since day 7 after photothrombosis, a hypointense signal was observed surrounding lesion area in photothrombosis mice, suggesting the formation of glia scar in photothrombosis mice which was also identified by immunostaining (Fig. 2). The MRI T2-w signal at 14 days after surgery can still be observed in photothrombosis model but hardly observed in MCAO model (Fig. 1 **a, b**). However in both models, Iba-1 expressing microglia were accumulated around the infarct lesion at day 14 after surgery with functional marker CD68 expression, prompting a functional activation status of microglia after 14 days ischemia (Fig. 2, see Additional file 1).

To determine neurodeficits in photothrombosis and MCAO model, a battery of behavioral tests on sensory-motor function were conducted, including mNSS score, the forelimb placing test, the adhesive-removing test, corner turning test, and rotarod test. As shown in Fig. 1c, the behavioral tests demonstrated that the greatest neurodeficits occurred from day 1 to day 3 in both photothrombosis and MCAO mice. Functional recovery started from day 7 post-stroke. Both photothrombosis and MCAO mice gradually and spontaneously recovered thereafter, but still exhibited neurodeficits up to 14 days after stroke as compared to sham control (Photothrombosis vs. sham, *n* = 10 per group, *p* < 0.01; MCAO vs. sham, *n* = 10 per group, *p* < 0.01; Fig. 1c). This result indicates that the impaired behavioral outcomes last up to 14 days after brain ischemia.

### Brain inflammation persists in photothrombosis and MCAO model during late-phase of brain ischemia

**Fig. 3:**
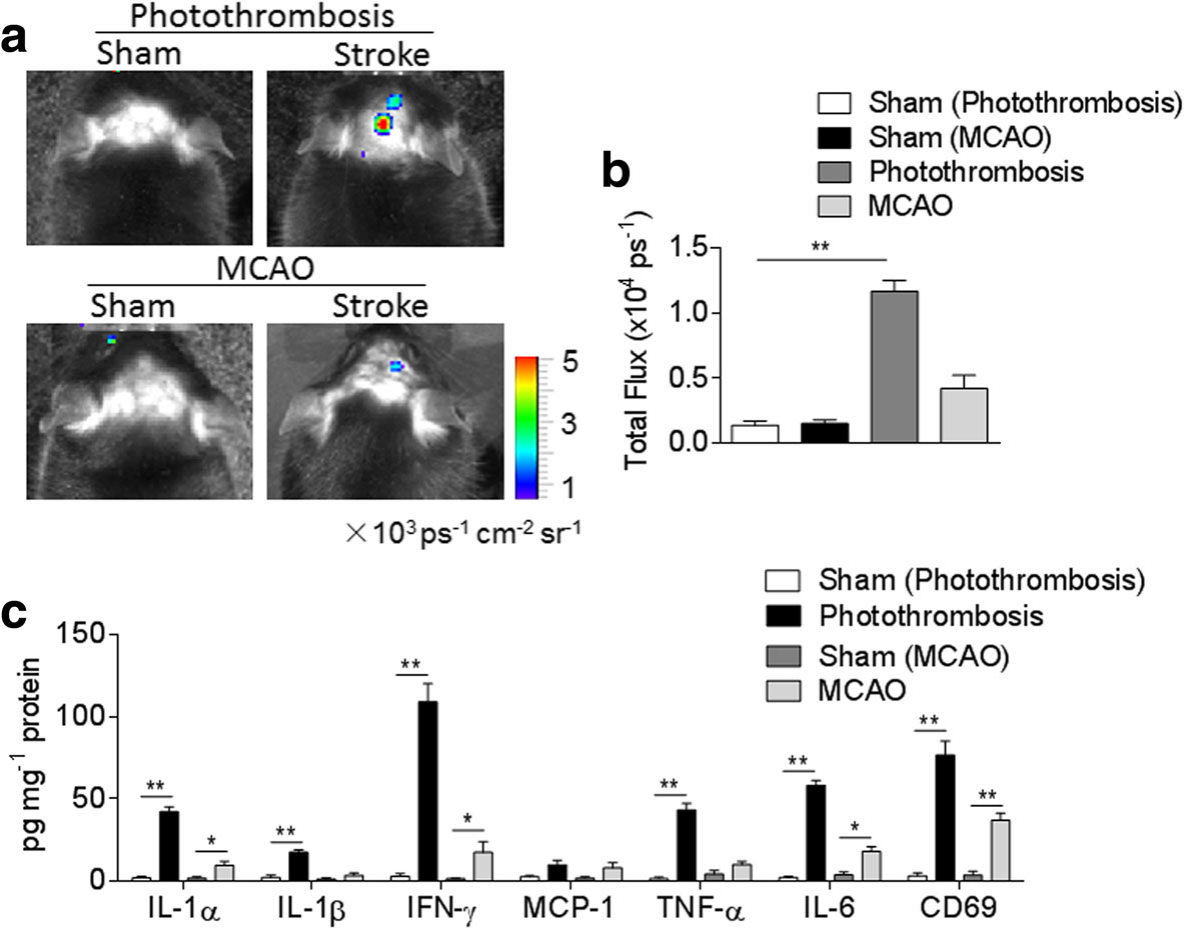
Production of ROS and inflammatory factors in the ischemic brain at day 14 after onset in mice. C57BL/6 (B6) mice were subjected to sham operation, photothrombosis, or 60 mins MCAO for 14 days. **a** At day 14 after surgery, bioluminescence images show ROS generation in photothrombosis and MCAO mice. **b** Quantification analysis showed ROS generation in photothrombosis and MCAO mice 14 days after ischemia. *n* = 6 mice per group. **c** At 14 days after ischemia, brain tissues were obtained from photothrombosis and MCAO mice. Brain tissues from sham-operated mice were used as control. Brain homogenates were prepared, and cytokine concentrations were measured by a customized Mouse Cytokines ELISA kit, including IL-1α, IL-1β, IFN-γ, MCP-1, IL-6, TNF-α, and CD69. Results shown are from three independent experiments with a pool of  4 mice per group. Error bars represent s.e.m.; *p < 0.05; **p < 0.01, sham vs. stroke group (photothrombosis/MCAO) by one-way ANOVA

To date, brain inflammation has been studied in many experimental models and human specimens during the acute phase of ischemic stroke. But the long-term features of inflammatory cells are still obscure. So we next investigated the inflammation status in CNS at late phase of brain ischemia in photothrombotic stroke model as well as MCAO model. As shown in Fig. 3, at 14 days after brain ischemia, a significantly higher level of reactive oxygen species (ROS; as one of the inflammatory parameters) was noted in photothrombosis mice and a slightly higher level of ROS was detected in MCAO mice as compared to sham mice (Photothrombosis vs. sham, *n* = 6 per group, *p* < 0.01; MCAO vs. sham, *n* = 6 per group, *p* > 0.05; Fig. 3a, b).

Using a customized mouse cytokine ELISA panel, we assessed the expression of pro-inflammatory cytokines in the brain homogenates of photothrombosis and MCAO mice 14 days after surgery. We found that both photothrombosis and MCAO for 14 days showed upregulation of pro-inflammatory cytokines release including IL-1α, IL-1β, IFN-γ, IL-6, TNF-α, and CD69 compared with sham control (Photothrombosis vs. sham, *n* = 4 per group, IL-1α, IL-1β, IFN-γ, IL-6, TNF-α, and CD69, *p* < 0.01; MCAO vs. sham, *n* = 4 per group, IL-1α, IFN-γ, IL-6, *p* < 0.05; CD69, *p* < 0.01), suggesting the brain inflammation persists until 14 days after brain ischemia (Fig. 3c).

### Lymphocytes infiltration and activation persist in late phase of brain ischemia in photothrombosis and MCAO mice

**Fig. 4:**
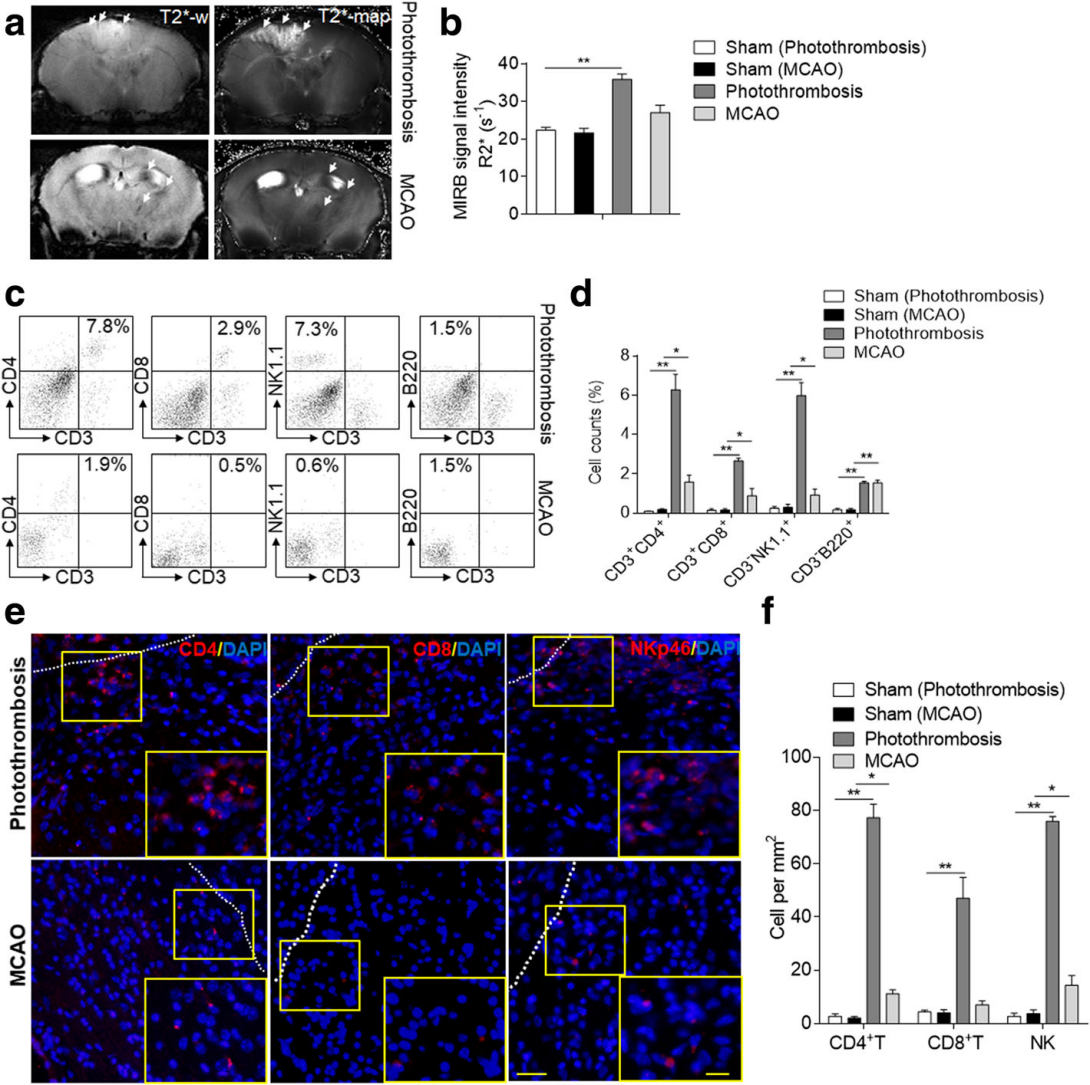
Lymphocyte infiltration persists in the ischemic brain at day 14 after onset in mice. **a** Lymphocytes were isolated from spleens of C57BL/6 (B6) mice and co-cultured with MIRB. 2 × 10^7^ MIRB-labeled cells were then adoptively transferred into Rag2^−/−^γc^−/−^ mice followed by sham or stroke procedures. MRI images show hypointensive dots that indicate MIRB-labeled lymphocytes in the ischemic brains of Rag2^−/−^γc^−/−^ mice. **b** Summarized results show signal of MIRB-labeled lymphocytes in mice receiving either sham or MCAO operations. *n* = 8 mice per group. **c** Dot plots of flow cytometry assay show CD4^+^ T, CD8^+^ T, NK, and B cells in single cell suspension from brains of photothrombosis- or MCAO-operated mice at 14 days after stroke. **d** Bar graphs summarize the cumulative data for quantifying CD4^+^ T, CD8^+^ T, NK, and B cell population from brains of photothrombosis- or MCAO-operated mice at 14 days after stroke. *n* = 8 mice per group. **e** At 14 day after surgery, representative immunostaining images show infiltrated CD4^+^ T, CD8^+^ T, and NK cells around ischemic lesion area in photothrombosis and MCAO mice. Red: CD4, CD8 or NKp46; blue: DAPI. Scale bars: 40 μm, 20 μm (inset). **f** Quantification analysis shows the infiltrated CD4^+^ T, CD8^+^ T, and NK cell counts from ipsilateral brain in photothrombosis and MCAO mice 14 days after ischemia. *n* = 5 mice per group. Error bars represent s.e.m.; *p < 0.05; **p < 0.01, sham vs. stroke group (photothrombosis/MCAO) by one-way ANOVA

As lymphocyte infiltration prominently contributes to brain inflammation during the delayed phase of ischemia, we further compared the brain lymphocyte infiltration in photothrombosis and MCAO mice at 14 days after brain ischemia. We used Molday ION Rhodamine B (MIRB), which is an ultra-small, super paramagnetic iron oxide particle (USPIO) of 35 nm that is not toxic for immune cells and can be visualized by MRI [20]. Lymphocytes were obtained from wild-type mice, labeled with MIRB in vitro, and then passively transferred into Rag2^−/−^γc^−/−^ recipient mice (lacking T, B, NK, and NKT cells). Using 7T-MRI, we non-invasively tracked inflammatory infiltration until 7 days after MCAO. We found that MIRB-labeled lymphocytes (visualized as hypo-intensive dots on MRI) could be tracked 7 days after surgery in both models, but with significantly stronger signal intensity in photothrombosis mice, indicating inflammatory infiltration of lymphocytes into the brain 7 days after stroke (Photothrombosis vs. sham, *n* = 8 per group, *p* < 0.01; MCAO vs. sham, *n* = 8 per group, *p* > 0.05; Fig. 4a, b).

Brain-infiltrating CD4^+^ T, CD8^+^ T, NK, and B cells can be observed both in photothrombosis and MCAO mice at 14 days after surgery. We found significantly increased CD4^+^ T, CD8^+^ T, NK cells in stroke models compared with sham mice. Comparing the two models, there are more CD4^+^ T, CD8^+^ T, NK cells in photothrombosis model than MCAO model, while no difference in B cell populations was observed between the two models (Photothrombosis vs. sham, *n* = 8 per group, CD4^+^ T, CD8^+^ T, NK, and B cells, *p* < 0.01; MCAO vs. sham, *n* = 8 per group, CD4^+^ T, CD8^+^ T, NK cells, *p* < 0.05; B cells, *p* < 0.01; Fig. 4c, d; Photothrombosis vs. sham, *n* = 5 per group, CD4^+^ T, CD8^+^ T, NK, *p* < 0.01; MCAO vs. sham, *n* = 5 per group, CD4^+^ T and NK cells, *p* < 0.05; Fig. 4e, f). This result suggested the lymphocyte infiltration can be observed in the delayed phase of brain ischemia, which might be implicated in long-term post-stroke injury.

**Fig. 5:**
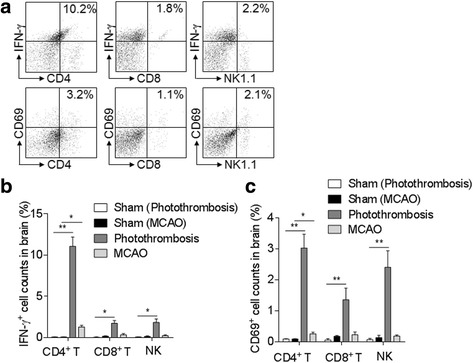
Brain-infiltrating lymphocytes express CD69 and IFN-γ at day 14 after brain ischemia in mice. **a** Flow cytometry dot plots show IFN-γ and CD69 expression in CD4^+^ T, CD8^+^ T, and NK cells in single cell suspension from brains of photothrombosis- or MCAO-operated mice at 14 days after stroke. **b**, **c** Bar graphs summarize the cumulative data for quantifying IFN-γ (**b**) and CD69 (**c**) expression in CD4^+^ T, CD8^+^ T, and NK cells from brains of photothrombosis- or MCAO-operated mice at 14 days after stroke. *n* = 8 mice per group. Error bars represent s.e.m.; **p < 0.01, sham vs. stroke group (photothrombosis/MCAO) by one-way ANOVA

**Fig. 6:**
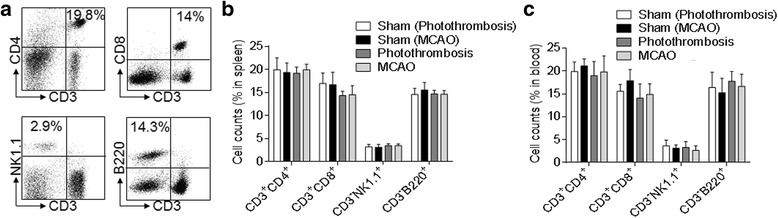
Assessment of peripheral lymphocyte subsets at day 14 after brain ischemia in mice. **a** Dot plots of flow cytometry show gating strategy of CD4^+^ T, CD8^+^ T, NK, and B cells in single cell suspension from spleens of photothrombosis- or MCAO-operated mice at 14 days after stroke. **b** Bar graphs summarize the cumulative data for CD4^+^ T, CD8^+^ T, NK, and B cell counts from spleens of photothrombosis- or MCAO-operated mice at 14 days after stroke. **c** Bar graphs summarize the cumulative data for CD4^+^ T, CD8^+^ T, NK, and B cell counts from blood of photothrombosis- or MCAO-operated mice at 14 days after stroke. *n* = 8 mice per group. Error bars represent s.e.m

Next, to further study the possible role of increased CD4^+^ T, CD8^+^ T, and NK cell infiltration in ischemic brain during the late phase, we compared the activation of these cells in brains of photothrombosis and MCAO mice with sham controls. We found that the activating lymphocyte marker CD69 and IFN-γ were over-expressed in both stroke models compared with sham mice, but more in photothrombosis mice as compared to MCAO mice at 14 days after surgery. Of note, in photothrombosis mice, IFN-γ expression was significantly increased in CD4^+^ T cells, which was not dramatic in CD8^+^ T or NK cells (IFN-γ: Photothrombosis vs. sham, *n* = 8 per group, CD4^+^ T cells, *p* < 0.01, CD8^+^ T and NK cells, *p* < 0.05; MCAO vs. sham, *n* = 8 per group, CD4^+^ T cells, *p* < 0.05; CD69: Photothrombosis vs. sham, *n* = 8 per group, CD4^+^ T, CD8^+^ T, and NK cells, *p* < 0.01; MCAO vs. sham, *n* = 8 per group, CD4^+^ T cells, *p* < 0.05; Fig. 5a–c). As IFN-γ is a critical cytokine in the mediation of Th1 responses that have been indicated as a key player to promote ischemic brain injury, these results indicate that the activation of infiltrated lymphocytes, especially IFN-γ-expressing CD4^+^ T cells, may be involved in the late phase of ischemic stroke. However, there is no significant difference in splenic and circulating lymphocyte populations between the sham and ischemic groups in the two models (Fig. 6).

### Rodent models mimic the inflammatory brain infiltration in ischemia stroke patients during delayed stage

**Fig. 7:**
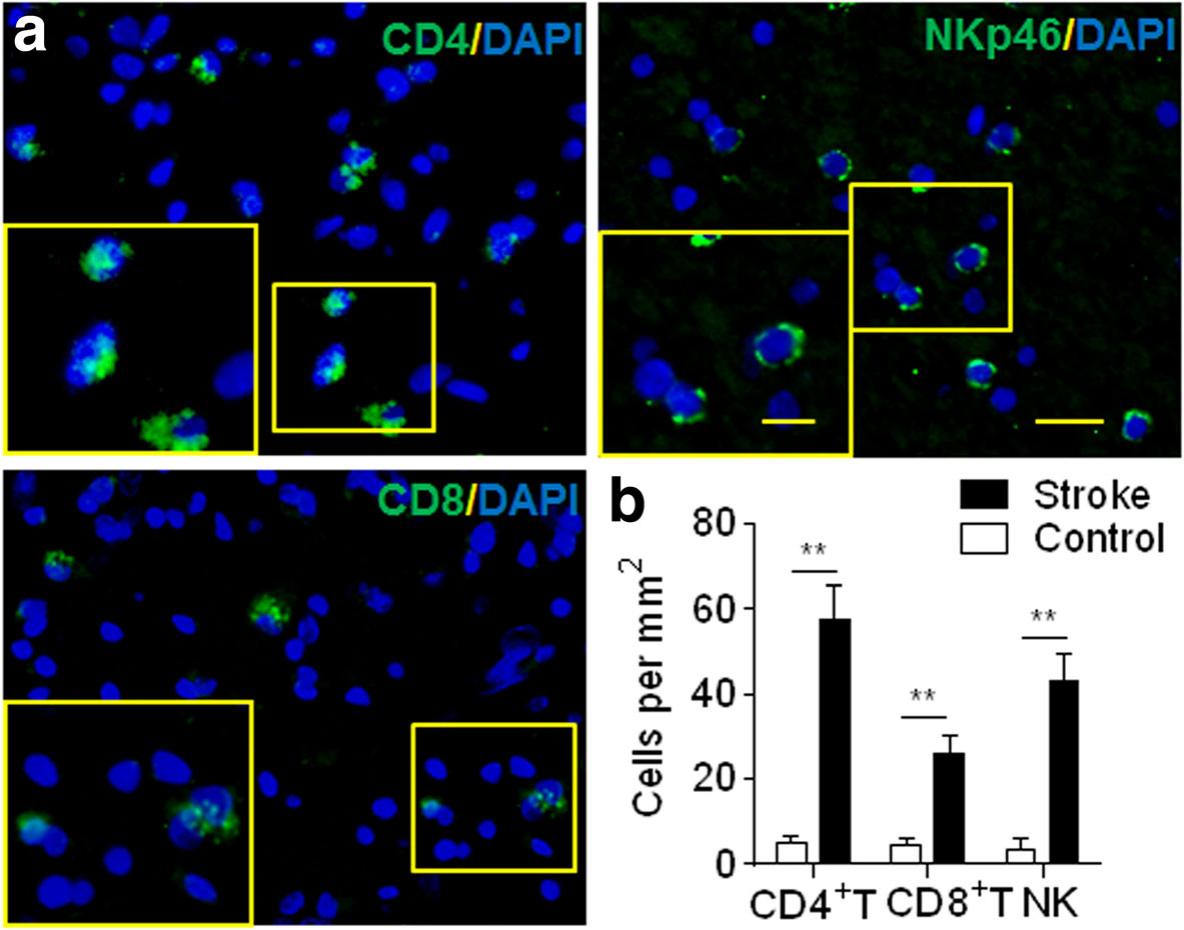
Lymphocyte infiltration persists in the brain of patients with ischemic stroke during delayed stage after onset. **a** Representative immunostaining images show CD4^+^ T, CD8^+^ T, and NK cells in brain perilesional area in an ischemic stroke patient. **b** Bar graph shows the quantification of CD4^+^ T, CD8^+^ T, and NK cells in controls and patients with ischemic stroke during later phases. *n* = 15 sections from five patients with ischemic stroke; *n* = 15 sections from five controls with non-neurological disease. Scale bars: 20 μm, 10 μm (inset). Error bars represent s.e.m.; **p < 0.01 sham vs. stroke patients by two-tailed unpaired Student’s *t* test

Ischemic stroke in human is a heterogeneous disorder with a complex pathophysiology at different stages, so it is not feasible to mimic all aspects of human stroke in one animal model. Here we examined brain infiltration of CD4^+^ T, CD8^+^ T, and NK cells in post-mortem brain sections from ischemic stroke patients who died within 7–14 days after stroke onset. Patients with ischemic stroke exhibited marked increase of T cells and NK cells around the infarct lesion area (Stroke vs. control, *n* = 15 sections, *p* < 0.01; Fig. 7). This result is paralleled with the lymphocyte infiltration in mice model, suggesting the persistence of inflammatory infiltration in both stroke patients and mice models, which provide the evidence to use murine models for mirroring the ischemic stroke patients, and for investigating immuno-inflammatory features at delayed stage of cerebral ischemia.

## Discussion

In this study, we demonstrate that lymphocyte infiltration persists during late stage of cerebral ischemia when brain infarcts become significantly decreased in two experimental stroke models. Together with the activation of infiltrating lymphocytes, we noted the increased ROS production, inflammatory factors release, and IFN-γ and CD69 upregulation in brain-infiltrated lymphocytes, suggesting that lymphocytes may retain their capability to impact the inflammatory microenvironment in the brain during the late stage of ischemia.

Infiltrating immune cells orchestrate the brain inflammatory environment by producing various effector molecules or inflammatory mediators [40]. In this study, we found that brain inflammation last to 14 days after brain ischemia in both models, and the photothrombotic mice elicited higher levels of inflammatory cytokines/chemokines in the brain and more lymphocyte infiltration from the periphery than MCAO mice during the late phase of stroke. This result may due to the different features of these two models: photothrombosis produces a permanent and persistent injury resulting from the production of free radicals and thrombus production in small vessels. In contrast, transient MCAO with an intraluminal occlusion produces a temporary occlusion of a main vessel that is resolved by reperfusion and collateral vessel perfusion. Furthermore, there is significant disruption of the blood-brain barrier surrounding photothrombotic regions for at least 7 and up to 14 days after injury [41], whereas the blood-brain barrier disruption is resolved within 4 days in MCAO [42], which indicates a more intact blood-brain barrier in MCAO mice by 14 days after injury. This factor may account for the differences observed in lymphocyte infiltration. Another possibility is the coagulation of peripheral blood during photothrombosis. In order to offset the possible confounding peripheral blood in the lesion, we also set up ET-1 focal stroke model with ET-1/L-NAME injection. We observed lymphocytes infiltration at day 14 after brain ischemia in ET-1 model, which supports our finding that inflammatory infiltration at late stage of brain ischemia (see Additional file 2). We further performed experiments to test how the coagulated blood within the cerebral vasculature affect observed ROS production and MIRB-labeled cell signal in photothrombosis model, by injecting lymphocytes before or immediately after photothrombosis procedure. As compared to the pre-stroke cell transfer, post-stroke transferred mice exhibit slightly reduced ROS production and MIRB signal but with no significance. This data indicates coagulation may produce artificial signal in the infarct region, but it may not significantly affect the lymphocyte infiltration-induced inflammation after brain ischemia (see Additional file 3). Our study suggests that lymphocytes may retain their capability to impact the inflammatory microenvironment in the brain during the late stage of ischemia. Cotrina ML et al. reported an enhanced inflammatory response and increased periphery infiltration within 7 days after photothrombotic stroke compared with MCAO, but did not study the long-term stroke outcome comparing the two models [43]. Our study also evaluated post-stroke neurological outcome, showing that in both models, neurodeficits still persist at late phase after brain ischemia. Vandeputte C et al. characterized the inflammatory response in photothrombotic stroke model by MRI [44], which is in line with our MRI results.

Early detrimental roles of lymphocytes such as T cells have been reported in ischemic stroke [16, 17]. Evidence suggests that CD4^+^ T and CD8^+^ T lymphocytes, but not B lymphocytes, contribute to the inflammatory thrombosis, brain injury, and neurological deficit in experimental stroke models [16, 17]. In an effort to identify the fate of brain-infiltrating lymphocytes, we measured the presence, activation, and functional status of these cells during the resolution phase of ischemic brain injury. The expression of the activation marker CD69 and cytokines such as IFN-γ by these lymphocytes suggest their competence to tune the inflammatory environment in the brain. Of interest, we found higher expression of IFN-γ in stroke mice, especially by CD4^+^ T cells. This finding suggests that T cells may be potentially involved in the process of tissue remodeling and impact disease outcome during the resolution phase of ischemic stroke. However, still unclear are how lymphocyte subsets such as T cells impact on brain ischemia-induced brain injury, what chronic factors and which neuro-immune pathways may be responsible. These aspects will certainly need to be investigated in future studies.

Although the precise temporal and spatial dynamics of lymphocyte response in human stroke may not entirely match that found in experimental stroke models because the overall disease time course is usually significantly longer in human stroke, it is notable that the persisted infiltration of lymphocytes were also seen in the brain sections from stroke patients during the delayed stage of ischemia. Comparing the two mice models with stroke patients, MCAO model is more similar in the vessel occluded and the reperfusion style expected in clinical cases, so better mirrors the ischemic stroke patient. While because of the lasting and distinct inflammatory infiltration, photothrombosis might be a useful animal model to study the brain inflammation during late phase of brain injury. This finding presents a need for further investigation to better understand the role of infiltrating lymphocytes during the late stage of cerebral ischemia.

## Conclusions

In summary, we demonstrate that lymphocyte infiltration persists during late-stage cerebral ischemia. The activation and expression of effector molecules in these cells suggests their potential involvement in the resolution phase of ischemic brain injury. These findings may provide a better knowledge base for future advanced studies to reveal the precise role of lymphocyte responses during late stage of stroke.

## Additional files

Additional file 1: Microglial phenotype at day 14 after photothrombosis and MCAO. C57BL/6 (B6) mice were subjected to sham operation, photothrombosis, or 60 mins MCAO for 14 days. At day 14 after surgery, mice were subjected to flow cytometry assessment. (**A**) Dot plots of flow cytometry assay show the gating strategy for microglia and CD68-expression. (**B**) Bar graphs summarize the cumulative data for quantifying microglia population and CD68-expression from brains of photothrombosis- or MCAO-operated mice at 14 days after stroke. *n* = 4 mice per group. *P < 0.05; **P < 0.01, sham vs. stroke (photothrombosis/MCAO) by one-way ANOVA. (DOCX 84 kb)

Additional file 2: Lymphocytes infiltration at day 14 after brain ischemia in ET-1 model. (**A**) ET-1 model induction. ET-1 and L-NAME were dissolved in sterile saline, and were delivered into the cortex by stereotaxic injection (AP +1.0, ML +1.0, DV -1.0, ET-1 at 1 μg and L-NAME at 2.7 μg). The ipsilateral common carotid artery were permanently occluded just prior to the ET-1 injection. (**B**) Lymphocyte infiltration at late stage of brain ischemia in mice subjected to ET-1 model. Dot plots of flow cytometry assay show CD4^+^ T, CD8^+^ T, NK, and B cells in single cell suspension from brains of sham (PBS injection) or ET-1/L-NAME injected mice at 14 days after procedures. (**C**) Bar graphs summarize the cumulative data for quantifying CD4^+^ T, CD8^+^ T, NK, and B cell counts from brains of ET-1 model at 14 days after stroke. *n* = 8 mice per group. Error bars represent s.e.m.; *P < 0.05; **P < 0.01, sham vs. ET-1 model by two-tailed unpaired Student’s *t* test. (DOCX 298 kb)

Additional file 3: ROS generation and lymphocytes infiltration in photothrombosis model with lymphocytes pre- or post-stroke transfer. Lymphocytes were isolated from spleens of C57BL/6 mice. 2 × 10^7^ isolated cells were then adoptively transferred into Rag2^−/−^γc^−/−^ mice followed by sham or photothrombosis procedures, or transferred immediately after procedure. (**A**) Imaging ROS activity in vivo. Bioluminescent images were captured for 1 min using the cooled IVIS imaging system (Xenogen IVIS-200) after luminol i.p. injection, to monitor the ROS generation in Rag2^−/−^γc^−/−^ photothrombosis brains. (**B**) Quantification and statistical analysis of the images. As compared to the pre-stroke cell transfer, post-stroke transferred mice exhibit slightly fewer ROS signal but with no significance. *n* = 3 mice per group. (**C**) Lymphocytes were isolated from spleens of C57BL/6 (B6) mice and co-cultured with MIRB. 2 × 10^7^ MIRB-labeled cells were then adoptively transferred into Rag2^−/−^γc^−/−^ mice followed by sham or stroke procedures, or transferred immediately after procedure. MRI was used to track MIRB-labeled lymphocytes in the ischemic brains of Rag2^−/−^γc^−/−^ mice. Bar graph shows MIRB signal in mice receiving either sham or photothrombosis model. MIRB signals can be observed in the ischemic brain of post-stroke transferred mice, with comparable intensity to the pre-transferred group. Error bars represent s.e.m.; *P < 0.05; **P < 0.01, sham vs. stroke by one-way ANOVA. (DOCX 257 kb)
