# Supplementary material for: Infiltration and persistence of lymphocytes during late-stage cerebral ischemia in middle cerebral artery occlusion and photothrombotic stroke models
Source: J Neuroinflammation. 2017 Dec 15;14:248. doi: 10.1186/s12974-017-1017-0 (PMC5732427; doi:10.1186/s12974-017-1017-0)
Supplement: Supplementary file 1 — Microglial phenotype at day 14 after photothrombosis and MCAO. C57BL/6 (B6) mice were subjected to sham operation, photothrombosis, or 60 mins MCAO for 14 days. At day 14 after surgery, mice were subjected to flow cytometry assessment. (A) Dot plots of flow cytometry assay show the gating strategy for microglia and CD68-expression. (B) Bar graphs summarize the cumulative data for quantifying microglia population and CD68-expression from brains of photothrombosis- or MCAO-operated mice at 14 days after stroke. n = 4 mice per group. *P < 0.05; **P < 0.01, sham vs. stroke (photothrombosis/MCAO) by one-way ANOVA. (DOCX 84 kb) [file 12974_2017_1017_MOESM1_ESM.docx]

**Additional file 1:**


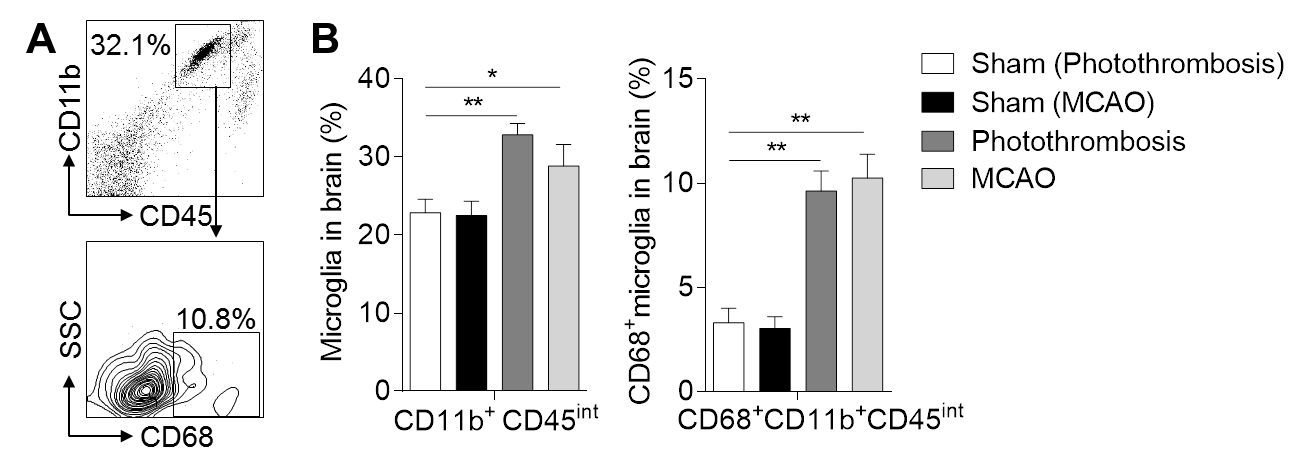


**Microglial phenotype at day 14 after photothrombosis and MCAO.** C57BL/6 (B6) mice were subjected to sham operation, photothrombosis or 60 mins MCAO for 14 days. At day 14 after surgery, mice were subjected to flow cytometry assessment. (**A**) Dot plots of flow cytometry assay show the gating strategy for microglia and CD68-expression. (**B**) Bar graphs summarize the cumulative data for quantifying microglia population and CD68-expression from brains of photothrombosis- or MCAO-operated mice at 14 days after stroke. n = 4 mice per group. *P < 0.05; **P < 0.01, sham vs. stroke (photothrombosis/MCAO) by one-way ANOVA.
