# Supplementary material for: Infiltration and persistence of lymphocytes during late-stage cerebral ischemia in middle cerebral artery occlusion and photothrombotic stroke models
Source: J Neuroinflammation. 2017 Dec 15;14:248. doi: 10.1186/s12974-017-1017-0 (PMC5732427; doi:10.1186/s12974-017-1017-0)
Supplement: Supplementary file 2 — Lymphocytes infiltration at day 14 after brain ischemia in ET-1 model. (A) ET-1 model induction. ET-1 and L-NAME were dissolved in sterile saline, and were delivered into the cortex by stereotaxic injection (AP +1.0, ML +1.0, DV -1.0, ET-1 at 1 μg and L-NAME at 2.7 μg). The ipsilateral common carotid artery were permanently occluded just prior to the ET-1 injection. (B) Lymphocyte infiltration at late stage of brain ischemia in mice subjected to ET-1 model. Dot plots of flow cytometry assay show CD4+ T, CD8+ T, NK, and B cells in single cell suspension from brains of sham (PBS injection) or ET-1/L-NAME injected mice at 14 days after procedures. (C) Bar graphs summarize the cumulative data for quantifying CD4+ T, CD8+ T, NK, and B cell counts from brains of ET-1 model at 14 days after stroke. n = 8 mice per group. Error bars represent s.e.m.; *P < 0.05; **P < 0.01, sham vs. ET-1 model by two-tailed unpaired Student’s t test. (DOCX 298 kb) [file 12974_2017_1017_MOESM2_ESM.docx]

**Additional file 2:**

**
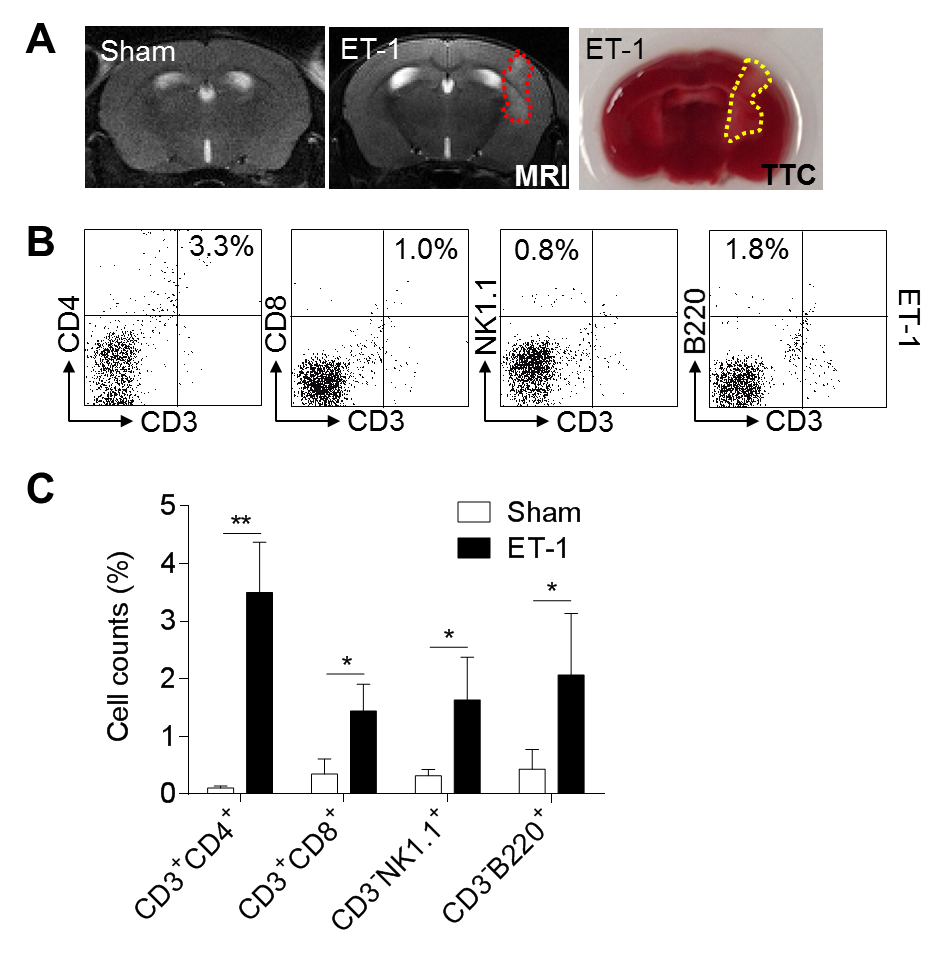
**

**Lymphocytes infiltration at day 14 after brain ischemia in ET-1 model.** (**A**) ET-1 model induction. ET-1 and L-NAME were dissolved in sterile saline, and were delivered into the cortex by stereotaxic injection (AP +1.0, ML +1.0, DV -1.0, ET-1 at 1µg and L-NAME at 2.7µg). The ipsilateral common carotid artery were permanently occluded just prior to the ET-1 injection. (**B**) Lymphocyte infiltration at late stage of brain ischemia in mice subjected to ET-1 model. Dot plots of flow cytometry assay show CD4^+^ T, CD8^+^ T, NK and B cells in single cell suspension from brains of sham (PBS injection) or ET-1/L-NAME injected mice at 14 days after procedures. (**C**) Bar graphs summarize the cumulative data for quantifying CD4^+^ T, CD8^+^ T, NK and B cell counts from brains of ET-1 model at 14 days after stroke. n = 8 mice per group. Error bars represent s.e.m.; *P < 0.05; **P < 0.01, sham vs. ET-1 model by two-tailed unpaired Student’s t-test.
