# Supplementary material for: Infiltration and persistence of lymphocytes during late-stage cerebral ischemia in middle cerebral artery occlusion and photothrombotic stroke models
Source: J Neuroinflammation. 2017 Dec 15;14:248. doi: 10.1186/s12974-017-1017-0 (PMC5732427; doi:10.1186/s12974-017-1017-0)
Supplement: Supplementary file 3 — ROS generation and lymphocytes infiltration in photothrombosis model with lymphocytes pre- or post-stroke transfer. Lymphocytes were isolated from spleens of C57BL/6 mice. 2 × 10^7^ isolated cells were then adoptively transferred into Rag2−/−γc−/− mice followed by sham or photothrombosis procedures, or transferred immediately after procedure. (A) Imaging ROS activity in vivo. Bioluminescent images were captured for 1 min using the cooled IVIS imaging system (Xenogen IVIS-200) after luminol i.p. injection, to monitor the ROS generation in Rag2−/−γc−/− photothrombosis brains. (B) Quantification and statistical analysis of the images. As compared to the pre-stroke cell transfer, post-stroke transferred mice exhibit slightly fewer ROS signal but with no significance. n = 3 mice per group. (C) Lymphocytes were isolated from spleens of C57BL/6 (B6) mice and co-cultured with MIRB. 2 × 10^7^ MIRB-labeled cells were then adoptively transferred into Rag2−/−γc−/− mice followed by sham or stroke procedures, or transferred immediately after procedure. MRI was used to track MIRB-labeled lymphocytes in the ischemic brains of Rag2−/−γc−/− mice. Bar graph shows MIRB signal in mice receiving either sham or photothrombosis model. MIRB signals can be observed in the ischemic brain of post-stroke transferred mice, with comparable intensity to the pre-transferred group. Error bars represent s.e.m.; *P < 0.05; **P < 0.01, sham vs. stroke by one-way ANOVA. (DOCX 257 kb) [file 12974_2017_1017_MOESM3_ESM.docx]

**Additional file 3:**

**
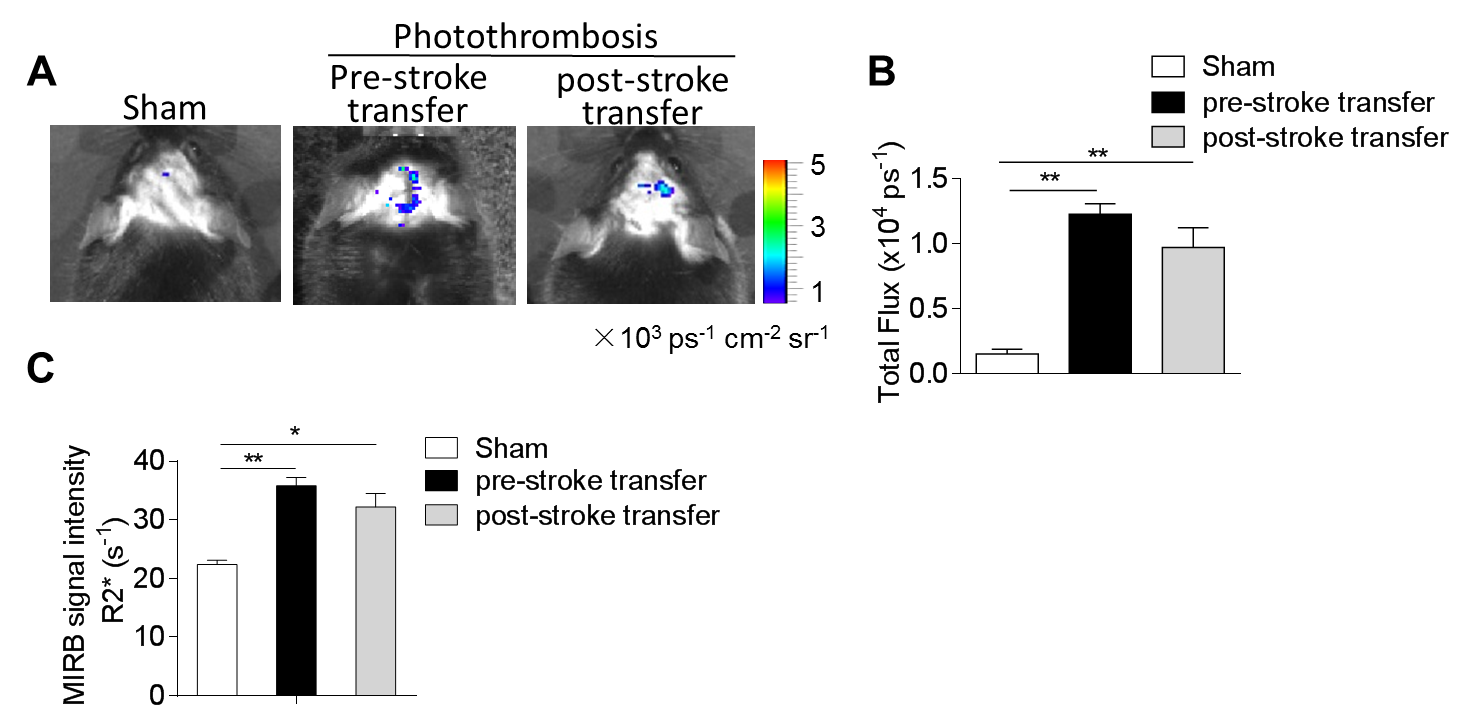
 ROS generation and lymphocytes infiltration in photothrombosis model with lymphocytes pre- or post-stroke transfer.** Lymphocytes were isolated from spleens of C57BL/6 mice. 2x10^7^ isolated cells were then adoptively transferred into Rag2^-/-^γc^-/-^ mice followed by sham or photothrombosis procedures, or transferred immediately after procedure. (**A**) Imaging ROS activity in vivo. Bioluminescent images were captured for 1 min using the cooled IVIS imaging system (Xenogen IVIS-200) after luminol i.p. injection, to monitor the ROS generation in Rag2^-/-^γc^-/-^ photothrombosis brains. (**B**) Quantification and statistical analysis of the images. As compared to the pre-stroke cell transfer, post-stroke transferred mice exhibit slightly fewer ROS signal but with no significance. n = 3 mice per group. (**C**) Lymphocytes were isolated from spleens of C57BL/6 (B6) mice and co-cultured with MIRB. 2x10^7^ MIRB-labeled cells were then adoptively transferred into Rag2^-/-^γc^-/-^ mice followed by sham or stroke procedures, or transferred immediately after procedure. MRI was used to track MIRB-labeled lymphocytes in the ischemic brains of Rag2^-/-^γc^-/-^ mice. Bar graph shows MIRB signal in mice receiving either sham or photothrombosis model. MIRB signals can be observed in the ischemic brain of post-stroke transferred mice, with comparable intensity to the pre-transferred group. Error bars represent s.e.m.; *P < 0.05; **P < 0.01, sham vs. stroke by one-way ANOVA.
